# Molecular Deceleration Regulates Toxicant Release to Prevent Cell Damage in Pseudomonas putida S16 (DSM 28022)

**DOI:** 10.1128/mBio.02012-20

**Published:** 2020-09-01

**Authors:** Hongzhi Tang, Kunzhi Zhang, Haiyang Hu, Geng Wu, Weiwei Wang, Xiongyu Zhu, Gongquan Liu, Ping Xu

**Affiliations:** a State Key Laboratory of Microbial Metabolism, Shanghai Jiao Tong University, Shanghai, People’s Republic of China; b Joint International Research Laboratory of Metabolic and Developmental Sciences, Shanghai Jiao Tong University, Shanghai, People’s Republic of China; c School of Life Sciences and Biotechnology, Shanghai Jiao Tong University, Shanghai, People’s Republic of China; d Zhejiang Center for Medical Device Evaluation, Zhejiang Medical Products Administration, Hangzhou, People’s Republic of China; Korea Advanced Institute of Science and Technology

**Keywords:** nicotine oxidoreductase, NicA2, pseudooxynicotine, crystal structure, molecular mechanism

## Abstract

The underlying molecular mechanisms of flavin-dependent amine oxidases remain relatively poorly understood, even though many of these enzymes have been reported. The nicotine oxidoreductase NicA2 is a crucial enzyme for the first step of nicotine degradation in Pseudomonas putida S16 (DSM 28022). Here, we present the crystal structure of a ternary complex comprising NicA2 residues 21 to 482, flavin adenine dinucleotide (FAD), and nicotine at 2.25 Å resolution. Unlike other, related structures, NicA2 does not have an associated diacyl glycerophospholipid, wraps its substrate more tightly, and has an intriguing exit passage in which nine bulky amino acid residues occlude the release of its toxic product, pseudooxynicotine (PN). The replacement of these bulky residues by amino acids with small side chains effectively increases the catalytic turnover rate of NicA2. Our results indicate that the passage in wild-type NicA2 effectively controls the rate of PN release and thus prevents its rapid intracellular accumulation. It gives ample time for PN to be converted to less-harmful substances by downstream enzymes such as pseudooxynicotine amine oxidase (Pnao) before its accumulation causes cell damage or even death. The temporal metabolic regulation mode revealed in this study may shed light on the production of cytotoxic compounds.

## Abstract (precis)

Flavin-dependent amine oxidases have received extensive attention because of their importance in drug metabolism, Parkinson’s disease, and neurotransmitter catabolism. However, the underlying molecular mechanisms remain relatively poorly understood. Here, combining the crystal structure of NicA2 (an enzyme in the first step of the bacterial nicotine degradation pathway in Pseudomonas putida S16 (DSM 28022)), biochemical analysis, and mutant construction, we found an intriguing exit passage in which bulky amino acid residues occlude the release of the toxic product of NicA2, in contrast to other, related structures. The selective product exportation register for NicA2 has proven to be beneficial to cell growth. Those seeking to produce cytotoxic compounds could greatly benefit from the use of such an export register mechanism.

## INTRODUCTION

Microbial cells are capable of ameliorating environmental exposures by metabolizing toxicants (1, 2). Examples include the use of bacteria to reduce toxicity at sites contaminated by polycyclic aromatic hydrocarbons (PAHs), dioxins, and nicotine (3–7). Microbes accomplish this detoxification by evolving complex enzymatic systems that are able to minimize self-damage from toxicants.

Large amounts of nicotine-containing wastes have been produced from tobacco processing and cigarette manufacture (8). If not properly handled, these nicotine-containing wastes can become serious environmental hazards, contaminating soil and freshwater (6–8). Conventional physical and chemical approaches for the removal of nicotine-containing wastes are relatively inefficient, so the use of microbiological organisms has emerged as a promising strategy for the degradation of nicotine and the neutralization of associated toxic by-products (6). Arthrobacter nicotinovorans and Pseudomonas putida are the bacteria known to efficiently degrade nicotine via, respectively, the pyridine and pyrrolidine pathways (6, 8). Both species can use nicotine and related catabolites as their sole sources of carbon and nitrogen.

In the first step of the nicotine metabolic pathway of the Gram-positive organism Arthrobacter nicotinovorans, the pyridine ring of nicotine is hydroxylated at the C-6 position to produce 6-hydroxy-l-nicotine (6HLN) or 6-hydroxy-d-nicotine (6HDN) (6). In the second step, the pyrrolidine ring of 6HLN or 6HDN is oxidized by 6-hydroxy-l-nicotine oxidase (6HLNO) or 6-hydroxy-d-nicotine oxidase (6HDNO) to form 6-hydroxy-*N*-methylmyosmine, which is then spontaneously hydrated to yield 6-hydroxy-pseudooxynicotine. The crystal structures of 6HLNO and 6HDNO have been determined, revealing the mechanism of recognition for their corresponding substrates, 6HLN and 6HDN (9–11).

**FIG 1:**
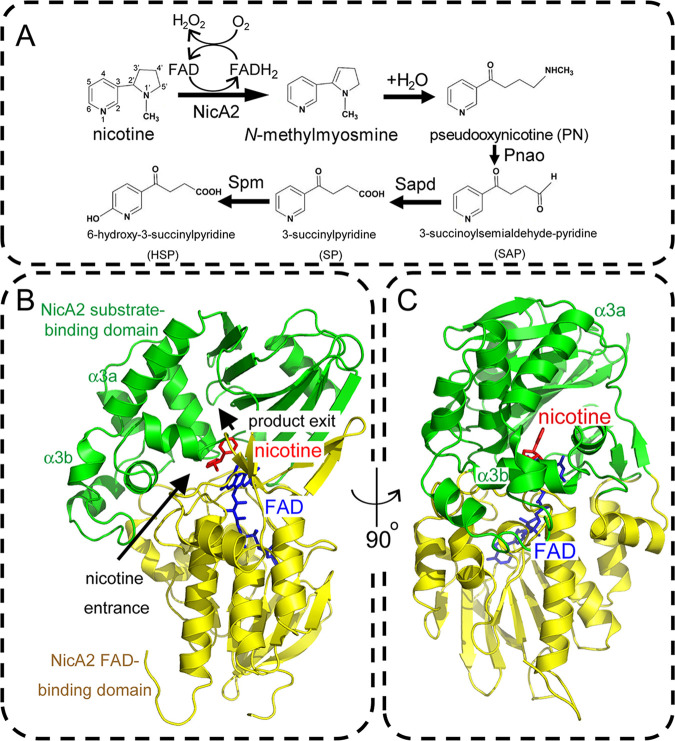
Overall structure of the nicotine oxidoreductase (NicA2) from Pseudomonas putida S16 in complex with the cofactor FAD and the substrate nicotine. (A) The upstream pathway of nicotine degradation in Pseudomonas putida S16. NicA2 catalyzes the dehydrogenation of the pyrrolidine moiety of nicotine to yield *N*-methylmyosmine, which is spontaneously hydrated to produce pseudooxynicotine (PN). PN is further converted to 3-succinoylsemialdehyde-pyridine (SAP) and then to 3-succinylpyridine (SP) by two sequential reactions catalyzed by Pnao and Sapd, respectively. SP is then hydroxylated by the trimeric SP monooxygenase (Spm) to form 6-hydroxy-3-succinylpyridine (HSP). (B) Crystal structure of the NicA2–FAD–nicotine ternary complex. The substrate-binding domain and the FAD-binding domain of NicA2 are color-coded green and yellow, respectively. FAD and nicotine are shown as sticks, color-coded blue and red, respectively. The entrance path for nicotine and the exit path for the reaction product PN are indicated by arrows. (C) The structure in panel B is rotated 90° counterclockwise. Note the kink between helices α3a and α3b, which breaks the continuity of these two helices.

Alternatively, in the nicotine degradation pathway of the Gram-negative organism Pseudomonas putida S16 (DSM 28022), the pyrrolidine ring of nicotine is dehydrogenated by the nicotine oxidoreductase NicA1 or NicA2 without prior hydroxylation of the pyridine ring (8, 12). The oxidation product *N*-methylmyosmine is then spontaneously hydrated to generate pseudooxynicotine (PN). In the NicA1- or NicA2-catalyzed reaction, flavin adenine dinucleotide (FAD) is required as a cofactor to couple the oxidation of nicotine with the reduction of oxygen to hydrogen peroxide (Fig. 1A). Pseudooxynicotine amine oxidase (Pnao) is capable of generating 3-succinoylsemialdehyde-pyridine (SAP) from PN. SAP is further metabolized to fumarate by dehydrogenation, hydroxylation, and oxidation reactions, two hydrolysis steps, and one *cis*-*trans* isomerization (12–18). The crystal structures of several enzymes involved in the later steps of nicotine degradation from P. putida strain S16 have been determined (19, 20). However, little structural information is available for any of the oxidoreductases that catalyze the earlier steps of this important pathway.

The nicotine oxidoreductase NicA2 is a crucial enzyme for nicotine degradation in P. putida S16. Deletion of *nicA2* results in the inability of the bacterium to grow (12). NicA2 from P. putida S16, 6HLNO and 6HDNO from A. nicotinovorans, and the nicotine oxidase NOX from *Pseudomonas* sp. strain HZN6 (21) belong to the family of flavin-dependent amine oxidases. Members of this family also include mammalian enzymes such as monoamine oxidase (MAO) A (22, 23), MAO B (24, 25), polyamine oxidase (PAO) (26), l-amino acid oxidase (LAAO) (27), and d-amino acid oxidase (DAAO) (28). Previous enzymatic studies on this family have generally favored a reaction mechanism involving direct hydride transfer. Recently, NicA2 from P. putida S16 has even been proposed as a resource for the development of novel nicotine addiction therapies (29, 30); the X-ray crystal structures of NicA2 alone and NicA2 in complex with nicotine have been reported at resolutions of 2.51 Å and 2.65 Å, respectively (31, 32).

In this study, we determined the crystal structure of NicA2 from P. putida S16 in complex with its cofactor FAD at 2.05 Å resolution, as well as that of the ternary complex of NicA2 with FAD and the substrate nicotine at 2.25 Å resolution. Notably, our structure reveals that the substrate nicotine is completely buried inside the active site pocket of NicA2 and that many bulky residues occlude the exit passage of PN.

We propose that the particularly occluded product exit passage of NicA2 was sculpted through evolution to accomplish a controlled release of the toxic reaction product PN. Such a controlled release would provide sufficient time for the downstream enzyme Pnao to degrade PN before considerable damage could be inflicted on the bacterial host. The temporal metabolic regulation of this register directly impacts important physiological processes.

## RESULTS AND DISCUSSION

To understand the molecular recognition mechanism of NicA2 for its cofactor FAD and the substrate nicotine, we first determined the crystal structure of selenomethionine (SeMet)-substituted NicA2 residues 21 to 482 [NicA2(21–482)] in complex with FAD at 2.05 Å resolution using the single-wavelength anomalous dispersion (SAD) method (Table 1). An N-terminal cut and SeMet substitution were performed to improve the quality and resolution of the crystals. Using this structure as a search model for molecular replacement, we then determined the crystal structure of the NicA2(21–482)–FAD–nicotine ternary complex at 2.25 Å resolution (Table 1). In both structures, there are two complexes in each asymmetric unit. However, as demonstrated by gel filtration chromatography (see Fig. S1 in the supplemental material), NicA2 behaves as a monomer in solution.

**TABLE 1:** Data collection and refinement statistics

Parameter^*a*^	Value for:	
	SeMet-NicA2(21–482) in complex with FAD	NicA2(21–482) in complex with FAD and nicotine
Data collection		
Space group	*P*4_1_2_1_2	*P*4_1_
Wavelength (Å)	0.97917	0.97924
Unit cell parameters (Å)	115.7, 115.7, 168.5	81.8, 81.8, 164.9
No. of molecules/asymmetric unit	2	2
Resolution range (Å) (outer shell)	100–2.05 (2.12–2.05)	50–2.25 (2.33–2.25)
Completeness (%) (outer shell)	99.8 (100.0)	95.7 (99.6)
Redundancy (outer shell)	8.1 (8.0)	3.7 (3.6)
Total observations	588,904	179,007
Unique reflections	72,473	49,191
*R*_merge_ (%) (outer shell)	14.4 (62.0)	7.6 (52.9)
*I*/σ*_I_* (outer shell)	25.8 (4.6)	15.7 (2.6)
Phasing		
Selenium sites found/expected	18/18	
Figure of merit	0.83	
Refinement		
Resolution range (Å)	100–2.05	50–2.25
R factor/*R*_free_ (%)	16.2/20.3	19.1/24.0
Overall B factor	24.7	53.6
RMSD bond lengths (Å)	0.006	0.005
RMSD bond angles (°)	1.118	0.904
No. of protein/substrate/water atoms in the final model modelmodelfinal model	6,880/0/553	6,892/24/283
Ramachandran plot (preferred, generally allowed, disallowed, %)	98.2, 1.6, 0.2	97.8, 1.9, 0.2

a *R*_merge_ = Σ*_h_*Σ*_i_* |*I_h_*_,_*_i_* – *I_h_*|/Σ*_h_*Σ*_i_ I_h_*_,_*_i_* for the intensity (*I*) of observation *i* of reflection *h*. R factor = Σ‖*F*_obs_| – |*F*_calc_‖/Σ|*F*_obs_|, where *F*_obs_ and *F*_calc_ are the observed and calculated structure factors, respectively. *R*_free_ = R factor calculated using 5% of the reflection data chosen randomly and omitted from the start of refinement. RMSD, root mean square deviations from ideal geometry. Data for the highest-resolution shell are shown in parentheses.

10.1128/mBio.02012-20.2 FIG S1 Elution profile of NicA2(21–482) from Superdex 200 gel filtration chromatography, which suggests that NicA2 exists as a monomer in solution. The elution volume for the standard molecular weight marker is indicated above the chromatogram. Download FIG S1, TIF file, 0.5 MB. Copyright © 2020 Tang et al. 2020 Tang et al. This content is distributed under the terms of the Creative Commons Attribution 4.0 International license.

The structure of NicA2 comprises a FAD-binding domain (residues 49 to 129, 250 to 338, and 417 to 482) that harbors the cofactor FAD and a substrate-binding domain (residues 130 to 249 and 339 to 416) associated with the substrate nicotine (Fig. 1B and C). The FAD-binding domain consists of a four-stranded β-sheet and a five-stranded β-sheet on one side, a couple of two-stranded β-sheets on the other side, and seven α helices and a 3_10_ helix in the middle (Fig. S2). The FAD cofactor adopts an extended conformation deeply inserted into the FAD-binding domain. FAD is tightly associated with NicA2 through extensive interactions between FAD and the FAD-binding domain. The substrate-binding domain of NicA2 can be divided into two subdomains: an all-α-helical S1 subdomain (residues 130 to 249) and an S2 subdomain (residues 339 to 416) consisting of a seven-stranded β-sheet, an α-helix, and a 3_10_ helix (Fig. S3). The nicotine molecule is bound midway between the S1 and S2 subdomains, with its pyrrolidine ring facing the FAD-binding domain.

10.1128/mBio.02012-20.3 FIG S2 Structure of the FAD-binding domain of NicA2 with the bound FAD cofactor. The FAD-binding domain of NicA2 is shown as a cartoon representation and colored in yellow, with secondary-structure elements labeled. FAD is shown as a stick representation, with carbon, nitrogen, oxygen, and phosphorus atoms colored in cyan, blue, red, and orange, respectively. Download FIG S2, TIF file, 2.3 MB. Copyright © 2020 Tang et al. 2020 Tang et al. This content is distributed under the terms of the Creative Commons Attribution 4.0 International license.

10.1128/mBio.02012-20.4 FIG S3 Structure of the substrate-binding domain of NicA2 with the bound substrate nicotine. The S1 and S2 subdomains of the substrate-binding domain of NicA2 are colored in green and magenta, respectively. Secondary-structure elements of NicA2 are labeled. Nicotine is shown as a stick representation, with carbon and nitrogen atoms colored in yellow and blue, respectively. Download FIG S3, TIF file, 1.6 MB. Copyright © 2020 Tang et al. 2020 Tang et al. This content is distributed under the terms of the Creative Commons Attribution 4.0 International license.

**FIG 2:**
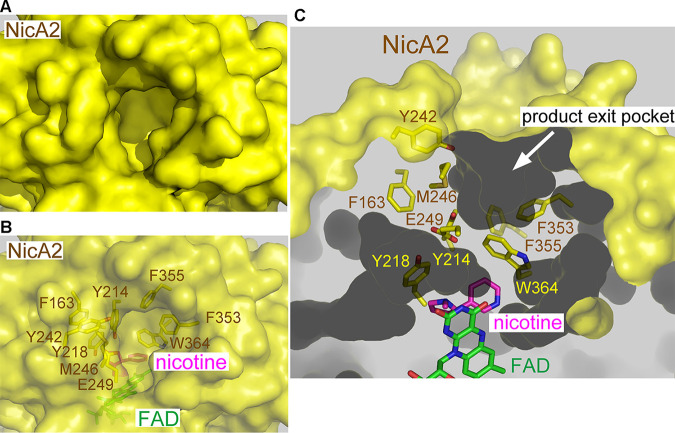
The exit passage for the reaction product PN within the NicA2 enzyme is occluded by several bulky residues. (A and B) NicA2 buries its substrate nicotine completely inside. NicA2 is shown as a surface representation. The nicotine molecule cannot be seen when viewed from outside the surface of NicA2 when its surface is set at 0% transparency (A) but can be seen to exist inside NicA2 when its surface is set at 40% transparency (B). (C) The exit passage of NicA2 for the reaction product PN is blocked by several bulky residues, including F163, Y214, Y218, Y242, M246, E249, F353, F355, and W364.

By analogy with what was reported for 6HLNO (11), the pathway of substrate entrance and product exit within the NicA2 molecule was inferred (Fig. 1B, arrows). Interestingly, the substrate nicotine is completely buried in the interior of NicA2 and is not accessible from the solvent when viewed from the substrate entry or product exit site (Fig. 2A). Only when the surface of NicA2 was set as transparent could the molecule nicotine be located deep inside the active-site pocket (Fig. 2B). When the product exit passage was investigated, it was found that this exit passage is blocked by many bulky residues, including W364, Y214, Y218, F355, F353, E249, F163, M246, and Y242 (Fig. 2B and C). We hypothesized that the molecular deceleration via the severely restricted product exit tunnel strongly hinders the release of the reaction product PN from NicA2.

**FIG 3:**
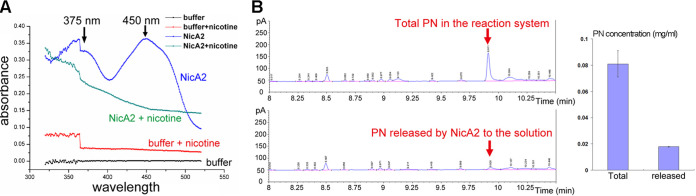
NicA2-catalyzed oxidation of nicotine is complete within seconds, whereas release of the reaction product PN from NicA2 is a much slower process. (A) NicA2-catalyzed oxidation of nicotine was complete within 10 s, as demonstrated by the UV-Vis spectroscopy assay. In the absence of nicotine, purified NicA2 protein exhibited absorbance peaks at 375 nm and 450 nm (blue curve), indicating that its associated FAD cofactor was in the oxidized state. On the other hand, 10 s after the addition of nicotine to NicA2, the absorbance peaks at 375 nm and 450 nm almost totally disappeared (green curve), suggesting that the associated FAD was reduced to FADH_2_ and the substrate nicotine was oxidized at the same time. The bump at 370 nm is due to the spectrometer’s switching of the running mode from UV scan to visible-light scan. (B) The release of the reaction product PN from NicA2 is a much slower process than the NicA2-catalyzed oxidation of nicotine. NicA2 was mixed with nicotine for 1.5 h, and then the NicA2 protein was removed by passing the mixture through a Ni^2+^-NTA affinity column. (Left) Gas chromatography was performed for the total mixture (total PN) (top) and the eluted fraction (free PN) (bottom) to measure the amounts of total PN generated in the reaction and PN released into solution from NicA2. (Right) Quantification of total and released PN. Three duplicate samples were set for each group. Error bars represent standard deviations.

NicA2 presents as yellow in solution, which is consistent with the presence of FAD as the cofactor. When nicotine was added to NicA2, the originally yellow NicA2 protein changed instantaneously to colorless. A UV-visible (UV-Vis) spectroscopy scan showed that the absorbance peaks at 375 nm and 450 nm of the purified NicA2 protein completely disappeared 10 s after the addition of nicotine (Fig. 3A). This indicated that within the 10 s, the associated FAD cofactor is reduced to FADH_2_, and the substrate nicotine is simultaneously oxidized to *N*-methylmyosmine and subsequently converted to PN.

However, when we measured the amount of the reaction product PN (both the total amount in the reaction system and the fraction released by NicA2 to the solution) by gas chromatography (GC), we found that, even after 1.5 h, only one-fourth of the PN produced in the reaction system was released to solution by NicA2 (Fig. 3B).

Therefore, the substrate nicotine is deeply buried inside the active-site pocket of NicA2, and the exit passage is too occluded to allow the quick release of PN. Although the NicA2-catalyzed oxidation of nicotine is fast and complete within seconds, the release of the reaction product PN from the active-site pocket of NicA2 is a time-consuming process, requiring hours to complete.

We reasoned that replacement of the bulky residues (such as phenylalanine, tyrosine, and tryptophan) blocking the exit passage of PN by amino acids with smaller side chains (such as alanine or valine) would facilitate the release of PN and enhance the catalytic turnover rate. To this end, we prepared a nine-residue point mutant construct of NicA2 (referred to as the 9-amino-acid [9AA] mutant) in which all the following mutations were performed: F163A, Y214A, Y218A, Y242A, M246A, E249A, F353V, F355V, W364V. When the enzymatic activities of wild-type (WT) NicA2 and the 9AA mutant were measured, the *k*_cat_ value of the 9AA mutant for *N*-methylmyosmine (23.2 × 10^−3^/s) was found to be 3.7 times higher than that of the WT enzyme (6.2 × 10^−3^/s). This result demonstrates that replacing the bulky residues at the product exit passage of NicA2 by amino acids with smaller side chains effectively increases the catalytic turnover rate of NicA2.

**FIG 4:**
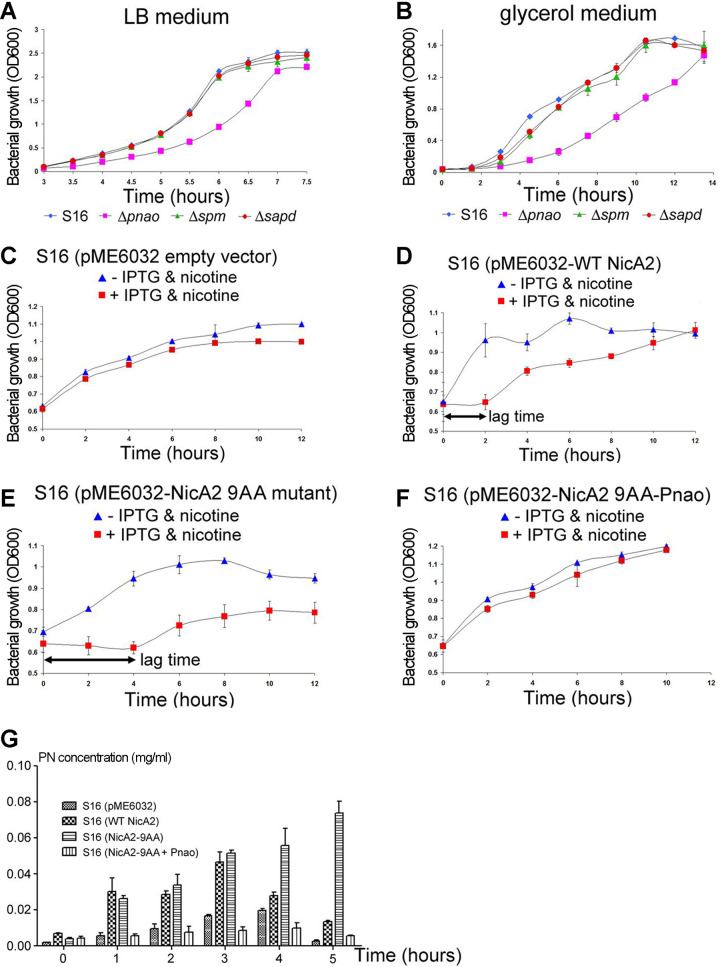
Knockout of the Pnao gene in P. putida S16 cells resulted in hindrance of bacterial cell growth in both nutrient-rich and nutrient-poor media, and mutations of the bulky residues at the PN exit passage of NicA2 to small-side-chain amino acids caused the bacteria to grow much more slowly, a defect that was rescued by the coexpression of Pnao. (A and B) Bacterial growth curves of WT (blue), ΔPnao (magenta), ΔSpm (green), and ΔSapd (red) strains in LB medium (A) or glycerol medium (B). (A) In the nutrient-rich LB medium, knockout of the *spm* or *sapd* gene did not have much adverse effect on the cell growth rate, whereas knockout of the Pnao gene caused the cells to grow much more slowly. The growth of WT P. putida S16 cells served as a control. (B) In the nutrient-poor glycerol medium, knockout of the Pnao gene caused the cells to grow much more slowly, while knockout of the Spm or Sapd gene did not have obvious effects. (C) The growth of a P. putida S16 cell culture transformed with the empty pME6032 plasmid was not much affected by the addition of IPTG and nicotine. (D) There was a lag time of 2 h for the growth of P. putida S16 cells overexpressing WT NicA2 upon the addition of IPTG and nicotine, but the cells could still grow up to an OD_600_ of ∼1.0 after 12 h of culturing. (E) The lag time for P. putida S16 cells overexpressing the 9AA mutant of NicA2 upon the addition of IPTG and nicotine was 4 h, twice as long as that for WT NicA2. Furthermore, the cell density (OD_600_) after 12 h of growth remained below 0.8. (F) When P. putida S16 cells were transformed with a pME6032 plasmid coexpressing both the 9AA mutant of NicA2 and the downstream enzyme Pnao, the addition of IPTG and nicotine did not affect the growth rate of bacterial cells. All experiments were performed in duplicate. (G) Overexpression of the 9AA mutant of NicA2 caused PN to accumulate, while coexpression of Pnao with the 9AA mutant of NicA2 prevented the accumulation. Shown are amounts of PN at different time points in bacterial cultures of P. putida S16 transformed with either the empty pME6032 plasmid, WT NicA2, the 9AA mutant of NicA2, or the 9AA mutant of NicA2 together with Pnao, as measured by gas chromatography. Three duplicate samples were set for each group. Error bars represent standard deviations.

We further postulated that deletion of the Pnao gene, whose corresponding protein decomposes PN (Fig. 1), would also lead to accumulation of PN and would thus be toxic to the bacterium. To evaluate this possibility, we monitored the growth rates of WT P. putida S16 and of strains in which Pnao, Spm, or Sapd gene had been deleted. In the nutrient-rich lysogeny broth (LB) medium, deletion of the Spm or Sapd gene did not have a noticeably deleterious effect on bacterial growth relative to that of WT P. putida S16 cells (Fig. 4A). In contrast, knocking out the Pnao gene resulted in a much lower cell growth rate in the same medium (LB) (Fig. 4A). These results were corroborated in the nutrient-poor glycerol medium (Fig. 4B). Therefore, the effects of Pnao gene knockout are equivalent to those of overexpression of the 9AA mutant of NicA2; both result in an increase in PN levels inside bacterial cells and have an adverse effect on the bacterial growth rate.

We hypothesized that the ability of NicA2 to control the release of PN might be favorable for the growth of P. putida S16 cells. To investigate this hypothesis, we monitored the growth of P. putida S16 strains transformed with WT NicA2 or the 9AA mutant of NicA2 in minimal medium, with or without the addition of isopropyl-β-d-thiogalactopyranoside (IPTG) and nicotine, respectively. The growth rate of P. putida S16 cells transformed with the control empty pME6032 plasmid was not affected by the addition of IPTG or nicotine alone (Fig. S4A) and was only moderately decreased when both IPTG and nicotine were added (Fig. 4C).

10.1128/mBio.02012-20.5 FIG S4 The addition of either IPTG alone or nicotine alone did not affect the growth of P. putida S16 cells transformed with WT NicA2 or the 9AA mutant of NicA2. (A) The growth rate of P. putida S16 cells transformed with the empty pME6032 plasmid was not affected by the addition of either IPTG or nicotine alone. (B) The growth rate of P. putida S16 cells overexpressing WT NicA2 was not affected by the addition of either IPTG or nicotine alone. (C) The growth rate of P. putida S16 cells overexpressing the 9AA mutant of NicA2 was not affected by the addition of either IPTG or nicotine alone. Three duplicate samples were set for each group, and the bars represent standard deviations. Download FIG S4, TIF file, 0.7 MB. Copyright © 2020 Tang et al. 2020 Tang et al. This content is distributed under the terms of the Creative Commons Attribution 4.0 International license.

When the pME6032 plasmid harboring WT NicA2 was transformed into P. putida S16, the cellular growth rate was not noticeably influenced by IPTG or nicotine alone (Fig. S4B). However, when both IPTG and nicotine were added, bacterial growth was inhibited. The cells resumed growing after a 2-h lag (Fig. 4D). Nevertheless, the cells were eventually able to reach an optical density at 600 nm (OD_600_) of ∼1.0 after 12 h of culturing (Fig. 4D).

Compared to the relatively moderate toxicity observed during overexpression of WT NicA2, overexpression of the 9AA mutant of NicA2 in the presence of IPTG and nicotine was more detrimental to bacterial growth. The lag period between the time points of induction with IPTG and nicotine and resumption of cell growth increased to 4 h, twice as long as that observed in cells transformed with WT NicA2. Moreover, these cells could not recover completely after the lag period, and the cell density (OD_600_) remained below 0.8 even after 12 h of cultivation (Fig. 4E). The addition of either IPTG or nicotine alone did not produce the same results (Fig. S4C).

To examine whether overexpression of WT NicA2 or the 9AA mutant of NicA2 was correlated with the PN level in bacterial culture, we measured the levels of PN in the culture medium using gas chromatography. Overexpression of WT NicA2 increased the amount of PN in the culture medium (Fig. 4G) over that observed in the control, and drastically higher levels were observed with overexpression of the 9AA mutant (Fig. 4G; note the 5-h time point).

Therefore, overexpression of WT NicA2 in P. putida S16 had a modest toxic effect on cell growth, as evidenced by a lag period between induction with IPTG and nicotine and the resumption of cell growth. Mutating the bulky residues at the exit passage of the reaction product PN to smaller residues enlarged the exit passage and enhanced the product release rate. This increase in the catalytic turnover rate of NicA2 was accompanied by growth hindrance in P. putida S16 cells and escalated levels of PN in the culture medium. This suggests that the reaction product PN is toxic for the bacteria and that WT NicA2 is equipped with a highly restricted PN exit passage to control its release and accumulation.

We reasoned that the lower growth rate of P. putida S16 cells overexpressing WT NicA2 and its 9AA mutant might have resulted from increased release of PN, causing damage to the bacterial cells. Given this assumption, overexpressing the downstream enzyme Pnao, which converts PN to SAP (Fig. 1), would likely reduce the amount of PN accumulating in bacteria and alleviate subsequent damage to the cells. Indeed, when Pnao was coexpressed along with the 9AA mutant of NicA2, a lag period for cell growth after induction with nicotine and IPTG was no longer observed (Fig. 4F). The bacteria grew as robustly as those transformed with the control empty plasmid (Fig. 4A and F).

When we measured the levels of PN in the culture by gas chromatography, we found that coexpression of Pnao together with the 9AA mutant of NicA2 decreased the amount of PN to background levels (Fig. 4G). Presumably, the PN produced is metabolized by the coexpressed Pnao as soon as it is released from the 9AA mutant of NicA2; hence, it is unable to accumulate within the bacterial cells.

In agreement with these results, overexpression of WT NicA2 also reduced the number of colonies of P. putida S16 on agar plates containing nicotine as the sole carbon source, while overexpression of the 9AA mutant of NicA2 had a more severe effect and further inhibited the growth of colonies. On the other hand, coexpression of Pnao with the 9AA mutant of NicA2 rescued the growth of bacteria by alleviating the toxic effects of PN (Fig. S5). These results further support our conjecture that the nicotine degradation product PN is disadvantageous for bacterial growth and that NicA2 has evolved a restricted PN exit passage to prevent its accumulation in the cells at an undesirable rate.

10.1128/mBio.02012-20.6 FIG S5 Overexpression of WT NicA2 also reduced the number of colonies of P. putida S16 on agar plates containing nicotine as the sole carbon source, while overexpression of the 9AA mutant of NicA2 had a more-severe effect and further inhibited the growth of colonies. On the other hand, coexpression of Pnao with the 9AA mutant of NicA2 rescued the growth of bacteria by alleviating the toxic effects of PN. Download FIG S5, TIF file, 3.0 MB. Copyright © 2020 Tang et al. 2020 Tang et al. This content is distributed under the terms of the Creative Commons Attribution 4.0 International license.

In summary, the product exit passage of NicA2 is blocked by nine bulky residues. This passage effectively controls the rate of release of the toxic reaction product PN and thus prevents its rapid accumulation in cells. This provides ample time for PN to be converted to less-harmful substances by downstream enzymes such as Pnao before it can accumulate and cause considerable damage to bacterial cells. Several practical applications for this type of selective release register mechanism immediately suggest themselves. Efforts to produce cytotoxic compounds, as in the initial development or bulked-up production of antibiotics or chemotherapy agents, could benefit from this combination of a buried active site and an occluded exit passage. It is easy to imagine the use of rapid targeted mutagenesis methods from synthetic biology to identify appropriate combinations of bulky residues to prevent or control the timing of the release of particular toxic reaction products.

## MATERIALS AND METHODS

### Materials.

L-(–)-Nicotine (≥99% purity) was obtained from Fluka Chemie GmbH (Buchs Corp., Buchs, Switzerland). FAD and NADH were obtained from Sigma-Aldrich (St. Louis, MO, USA). ^18^O_2_ was from the Shanghai Research Institute of Chemical Industry. Dihydropyran (DHP) was from SynChem OHG (Kassel Corp., Kassel, Germany). 6-Hydroxy-3-succinylpyridine (HSP) was isolated and purified from the culture broth of strain S16 (8). Source 30Q, Source 15ISO, and Sephadex G-25, Mono Q 5/50 GL, and Sephacryl S-200 HR columns were from GE Healthcare (Uppsala, Sweden).

### Protein expression and purification.

The DNA fragments encoding full-length (FL) wild-type (WT) NicA2 and NicA2(21–482) were cloned either into the pET28a vector (Novagen) with a 6×His tag at the C-terminal end or into the Escherichia coli-P. putida S16 shuttle vector pME6032 by standard molecular cloning procedures. The 20 amino acids at the N terminus of NicA2 were predicted to form loose loops, irrelevant to enzyme activity, and were cut to improve the quality of the crystals.

The gene encoding the 9AA mutant (with the F163A, Y214A, Y218A, Y242A, M246A, E249A, F353V, F355V, and W364V mutations) of FL NicA2 was chemically synthesized and cloned into the pET28a or pME6032 vector.

Various constructs of NicA2 were overexpressed in Escherichia coli strain BL21(DE3). Cells were grown at 37°C to an OD_600_ of 0.8 and were induced with 200 μM IPTG overnight at 16°C. Cells were harvested by centrifugation at 12,000 rpm, and the cell pellet was resuspended in binding buffer (25 mM Tris-HCl [pH 8.0], 300 mM NaCl, and 20 mM imidazole) and lysed with a cell homogenizer (JNBIO). The cell lysate was centrifuged, and the resulting supernatant was purified using Ni^2+^-nitrilotriacetic acid (NTA) affinity chromatography (Qiagen) and Superdex 200 gel filtration chromatography (GE Healthcare). The gel filtration buffer contained 25 mM Tris-HCl (pH 8.0), 150 mM NaCl, and 5 mM dithiothreitol (DTT). Peak fractions were combined and concentrated to 12 mg/ml, flash-frozen in liquid nitrogen, and stored at –80°C.

Selenomethionine (SeMet)-substituted NicA2 was overexpressed using the methionine-autotrophic E. coli strain B834(DE3) cultured in M9 minimal medium and was purified using the same procedure as for the native protein, except that 10 mM DTT was used in the Superdex 200 gel filtration buffer.

### Crystallization.

The hanging-drop vapor diffusion method was employed for crystallization by mixing 2 μl protein with 1 μl reservoir solution and equilibrating against 200 μl reservoir solution in each well. Crystals of the NicA2–FAD complex and the SeMet–NicA2–FAD complex were obtained from condition no. 36 of SaltRx2 (Hampton Research), which consists of 1.4 M ammonium tartrate and 0.1 M Tris (pH 8.5). Crystals were grown to full size within 3 weeks at 14°C. The cryoprotectant used was 1.4 M ammonium tartrate, 0.1 M Tris (pH 8.5), and 20% (vol/vol) glycerol.

Crystals of the NicA2–FAD–nicotine complex were prepared by soaking crystals of the NicA2–FAD complex in cryoprotectant buffer, supplemented with 40 mM nicotine and 50 mM sodium dithionite, for about 5 s before harvesting with nylon cryoloops (Hampton Research) and flash-cooling in liquid nitrogen.

### Data collection and processing.

Crystal diffraction data sets of the SeMet–NicA2–FAD complex and the NicA2–FAD–nicotine complex were collected at the BL17U1 beamline from the Shanghai Synchrotron Radiation Facility (SSRF), Shanghai, China, by using an ADSC Quantum 315r CCD area detector. All data were processed and scaled using the HKL2000 program (33). Crystals of the SeMet–NicA2–FAD complex belonged to the tetragonal space group *P*4_1_2_1_2, while the NicA2–FAD–-nicotine crystals belonged to the tetragonal space group *P*4_1_. There are four monomers per asymmetric unit in both the crystals of the SeMet–NicA2–FAD complex and the crystals of the NicA2–FAD–nicotine complex. Data collection statistics are summarized in Table 1.

### Structure determination.

The structure of the SeMet–NicA2–FAD complex was determined using the single-wavelength anomalous dispersion (SAD) method present in the Autosol and Autobuild modules of PHENIX (34). All 18 selenium sites in the asymmetric unit were correctly located. After iterative model building using Coot (35) and refinement using the CCP4 program REFMAC5 (36, 37), the final model had *R*/*R*_free_ values of 16.2%/20.3% and included residues 32 to 482 of NicA2.

The structure of the NicA2–FAD–nicotine complex was determined using the molecular replacement method present in the CCP4 program Phaser (36, 38), with the refined structure of the SeMet–NicA2–FAD complex as a searching model. After model building using Coot (35) and refinement using REFMAC5 (36, 37), the final model had *R*/*R*_free_ values of 19.1%/24.0%. The refinement statistics are listed in Table 1.

### UV-Vis spectroscopy.

For the UV-Vis spectroscopic measurement, NicA2 protein was adjusted to 10 mg/ml using 25 mM Tris-HCl (pH 8.0) containing 2 mM β-mercaptoethanol. A UV-Vis scan from 300 nm to 600 nm was performed, and the absorbance at 450 nm for the nondenatured NicA2 and NicA2–nicotine samples was used to determine whether the FAD was in the oxidized or reduced form, while an equal sample volume of 25 mM Tris-HCl (pH 8.0) containing 2 mM β-mercaptoethanol was used as a blank.

### PN extraction and GC analysis.

To measure the amount of pseudooxynicotine (PN) released in the *in vitro* assay using purified protein, NicA2 was incubated at 25°C with an equimolar amount of nicotine for 1.5 h, and then the NicA2 protein was removed by passing the mixture through the Ni^2+^-nitrilotriacetate agarose column. The flowthrough fraction containing the free PN was collected and was then dried by vacuum freezing after being stored at –80°C for at least 3 h.

To measure the amount of PN released to the culture in the bacterial cell-growing assay, bacteria were centrifuged to the cell pellet, and the supernatant (2 ml) was evaporated to dryness using the same method as described above.

All samples for PN quantification were then dissolved in 200 μl benzyl alcohol–chloroform at a 1:1,000 ratio, with 20 μl of 1 M Na_2_CO_3_ added to adjust the pH to 7.0. The Agilent 7890 GC oven contained two capillary columns. The oven was kept at 60°C for 1 min at first, followed by an increase of 5°C/min to 165°C, then at 25°C/min to a final temperature of 280°C, which was held for 14 min. The splitless injection technique was used. The volume of sample injected was 1 μl. PN was identified by its retention time using the commercial compound as a standard. Quantification was carried out using the measured relative areas under each peak. Experiments were performed in triplicate.

### Enzymatic activity assays for NicA2.

NicA2 activity was determined by liquid chromatography-mass spectrometry (LC-MS) using an Agilent 1290 infinity liquid chromatograph with 6230 quadrupole mass spectrometry. After incubation at room temperature for 20 min, 2.5-fold the total volume of acetonitrile was added to quench the reaction. A 20-μl portion of each sample was injected into a Poroshell 120 EC-C8 column (4.6 by 100 mm; 1.8 μm; Agilent Technologies) at a constant flow rate of 0.2 ml/min and was subjected to a gradient as reported previously (29). MS analysis was performed in the Turbo ion spray mode with positive ions. The data for WT NicA2 or the 9AA mutant at various concentrations of nicotine were fitted to obtain the *k*_cat_ value and *K_m_*.

### Electroporation and bacterial cell growth assays.

P. putida S16 was transformed by electroporation using the following conditions: 0.5 to 1 mg plasmid DNA was added to 100 μl electrocompetent cells of P. putida S16, and the mixture was electroporated at 12 kV/cm, 200 Ω, and 25 μF using a Bio-Rad Gene Pulser Xcell system (Bio-Rad Laboratories, Hercules, CA). After incubation at 30°C in a shaker (200 rpm) for 1.5 h, the transformants were plated onto LB agar plates with tetracycline (20 μg/ml) and nicotine (1 mg/ml). A single colony of transformants was randomly selected from the plates, and a 1:100 dilution of a fresh overnight culture was inoculated into 50 ml minimal medium in a 250-ml flask (performed in triplicate).

For the bacterial cell growth assays, transformants were grown at 30°C in a basal mineral medium with 1 mg/ml nicotine as the sole carbon and nitrogen source. When the OD_600_ of the cultures reached 0.6 to 0.7, 3 mg/ml nicotine and 0.8 mM IPTG were added. The cultures were then grown for an additional 12 h at 30°C. The growth of each transformant was monitored every 2 h.

For the cell growth assay in petri dishes, after the transformants were cultured for 2 h, equivalent amounts of cells were plated onto the solid medium of 1.5% (w/v) agar powder that contained the same ingredients as the liquid medium described above.

### Bacterial cell growth assay for coexpressing the 9AA mutant of NicA2 with Pnao.

For the bacterial cell growth assay of the 9AA mutant coexpressed with Pnao, the combined genes encoding the 9AA mutant of NicA2 and Pnao with an intervening Shine-Dalgarno sequence (AAGGAGATATACC) between them were PCR amplified and then cloned into the pME6032 shuttle vector. P. putida S16 was transformed by electroporation according to the protocol presented above, and the growth of P. putida S16 cells coexpressing NicA2-9AA and Pnao was monitored by measuring OD_600_ every 2 h.

### Bacterial cell growth assay of P. putida S16 with various genes deleted.

P. putida strain S16 cells or those with the *pnao*, *sapd*, or *spm* gene deleted (13) were cultured in LB medium with 1 mg/ml nicotine. The cell density of each transformant was measured every half hour. As a parallel experiment, the cells were also cultivated in a glycerol medium containing 1 g/liter glycerol, 1 g/liter (NH_4_)_2_SO_4_, 13.3 g/liter K_2_HPO_4_·3H_2_O, 4 g/liter KH_2_PO_4_, 0.2 g/liter MgSO_4_·7H_2_O, and 0.5 mg/liter trace element solution, with an initial pH of 7.0. The trace element solution contained the following materials per liter of 0.1 M HCl: 0.05 g CaCl_2_·2H_2_O, 0.05 g CuCl_2_·2H_2_O, 0.008 g MnSO_4_·H_2_O, 0.004 g FeSO_4_·7H_2_O, 0.1 g ZnSO_4_, 0.1 g Na_2_MoO_4_·2H_2_O, and 0.05 g Na_3_WO_4_·2H_2_O. The cell density of each transformant was measured every hour.

### Accession number(s).

The atomic coordinates and structure factors of the selenomethionine-substituted NicA2–FAD complex and the NicA2–FAD–nicotine complex have been redeposited and released in the Protein Data Bank with accession codes 7C4A (released 3 June 2020) and 7C49 (released 3 June 2020). The same data were previously deposited as 5GWC (deposited 9 September 2016) and 5GWH (deposited 11 September 2016), respectively.
