# Supplementary figures and images for: Molecular Deceleration Regulates Toxicant Release to Prevent Cell Damage in Pseudomonas putida S16 (DSM 28022)
Source: mBio. 2020 Sep 1;11(5):e02012-20. doi: 10.1128/mBio.02012-20 (PMC7468206; doi:10.1128/mBio.02012-20)

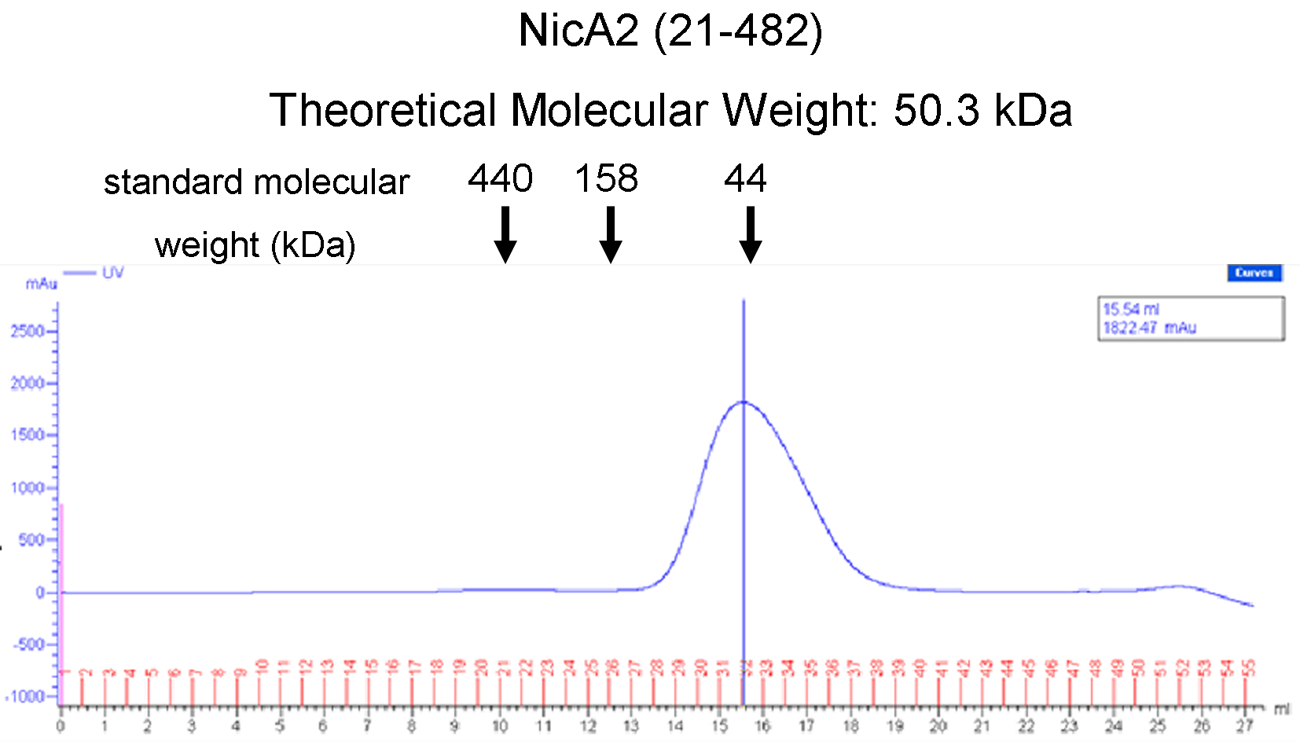

Supplement: FIG S1 [file mBio.02012-20-sf001.tif]

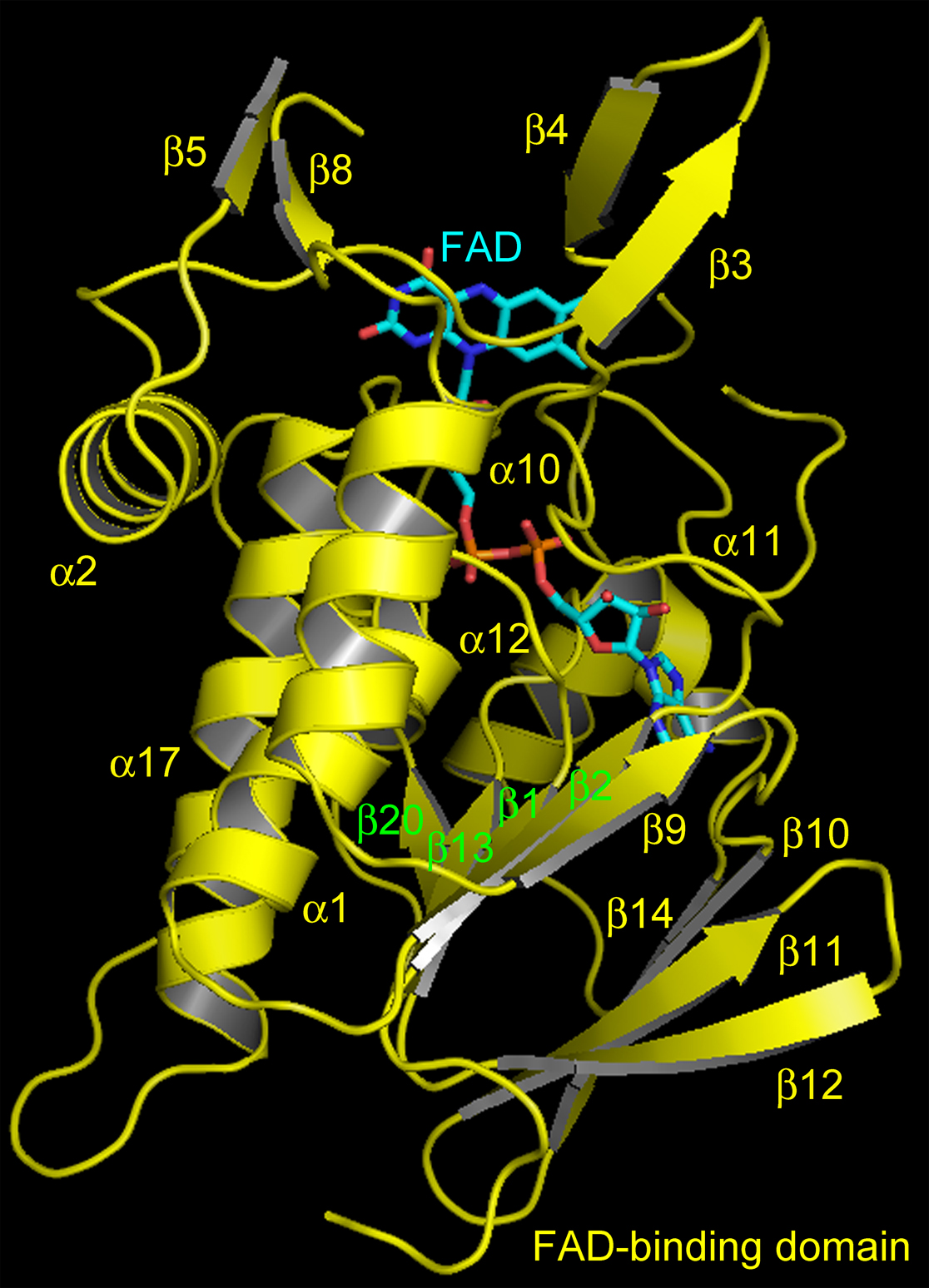

Supplement: FIG S2 [file mBio.02012-20-sf002.tif]

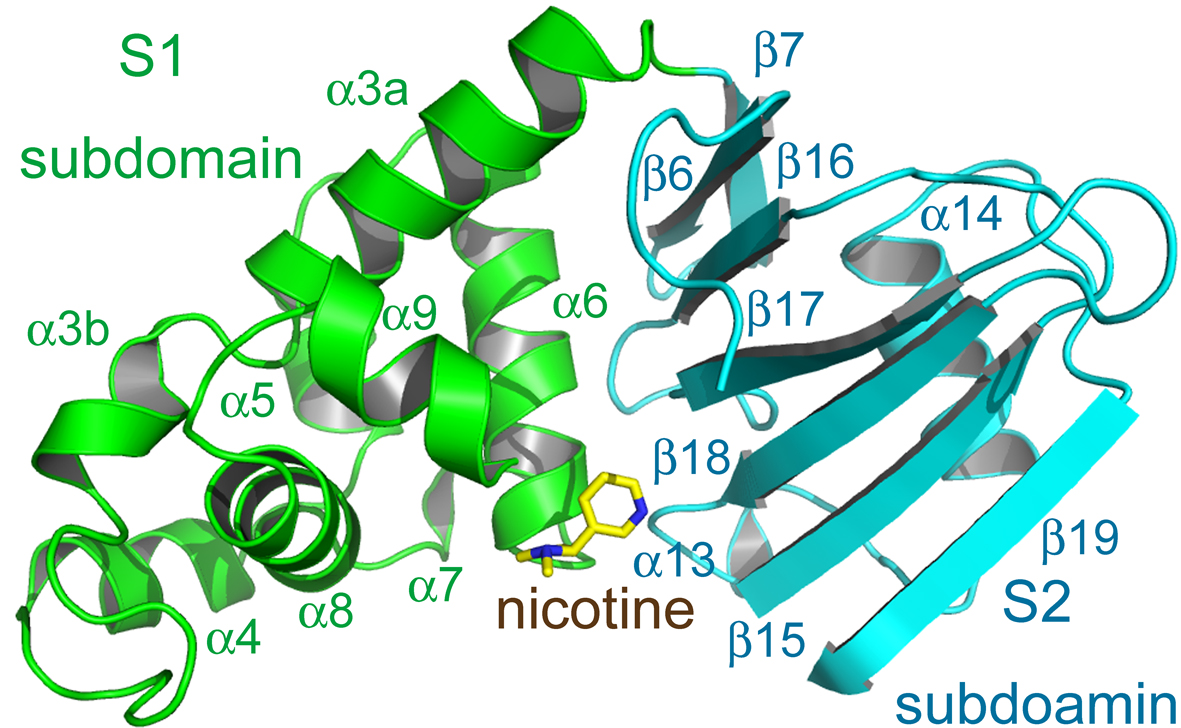

Supplement: FIG S3 [file mBio.02012-20-sf003.tif]

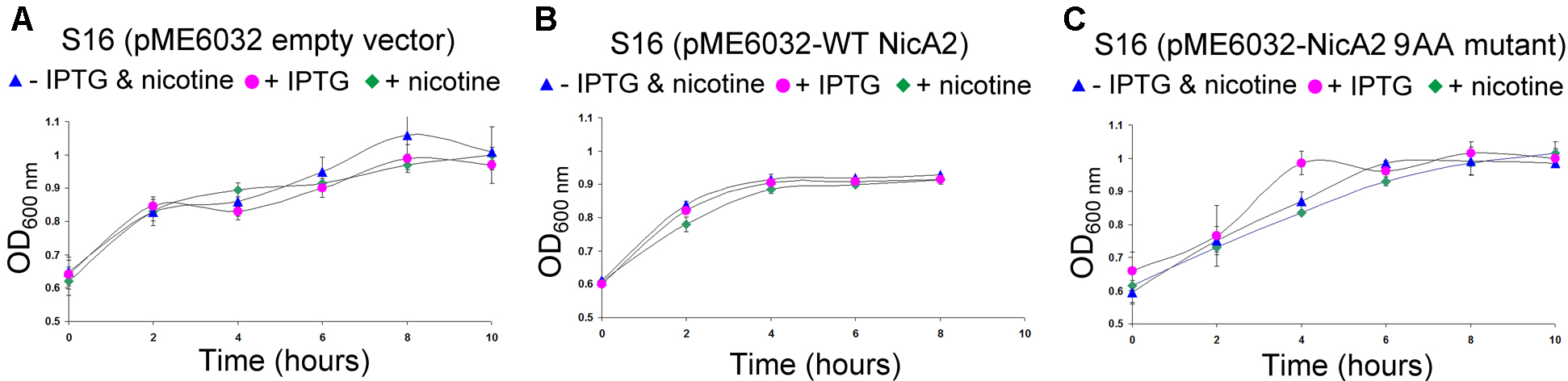

Supplement: FIG S4 [file mBio.02012-20-sf004.tif]

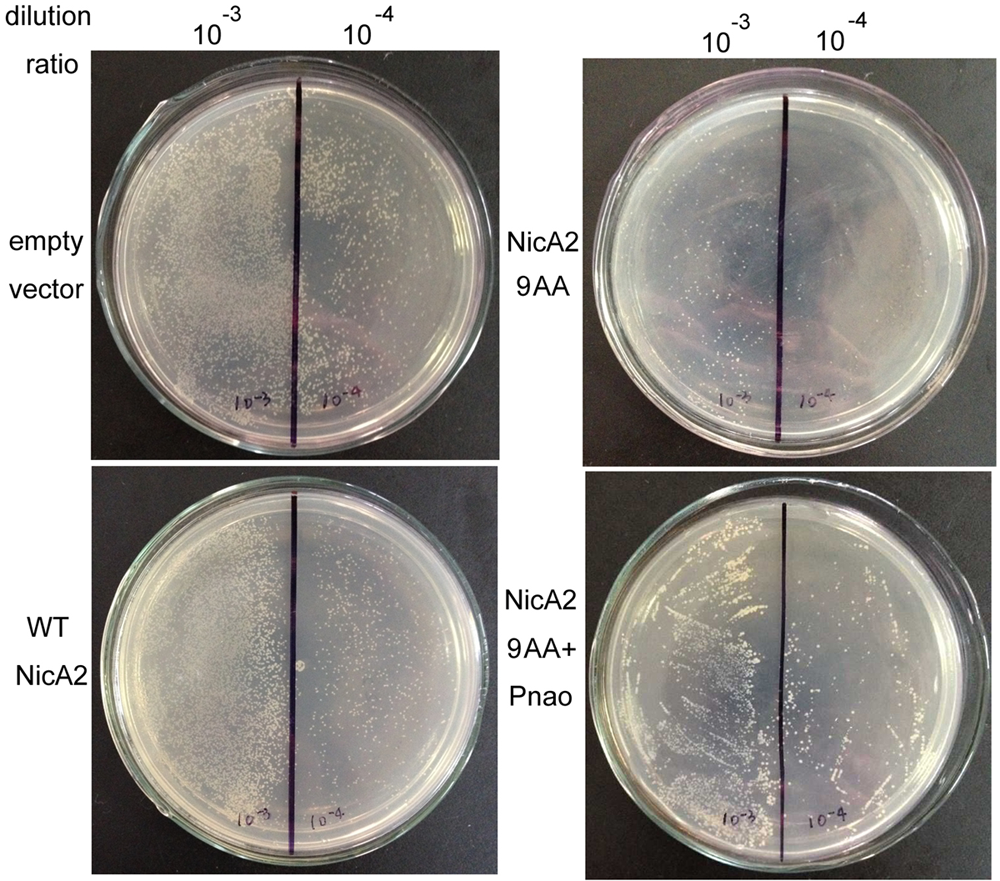

Supplement: FIG S5 [file mBio.02012-20-sf005.tif]
